# Brain size as a driver of avian escape strategy

**DOI:** 10.1038/srep11913

**Published:** 2015-07-03

**Authors:** Diogo S. M. Samia, Anders Pape Møller, Daniel T. Blumstein

**Affiliations:** 1 Laboratory of Theoretical Ecology and Synthesis, Department of Ecology, Federal University of Goiás, CP. 131, 74001-970 Goiânia, Brazil; 2 Laboratoire d’Ecologie, Systématique et Evolution, CNRS UMR 8079, Université Paris-Sud, Bâtiment 362, F-91405 Orsay Cedex, France; 3 Department of Ecology and Evolutionary Biology, University of California, 621 Young Drive South, Los Angeles, California, 90095-1606, USA

## Abstract

After detecting an approaching predator, animals make a decision when to flee. Prey will initiate flight soon after detecting a predator so as to minimize attentional costs related to on-going monitoring of the whereabouts of the predator. Such costs may compete with foraging and other maintenance activities and hence be larger than the costs of immediate flight. The drivers of interspecific variation in escape strategy are poorly known. Here we investigated the morphological, life history and natural history traits that correlate with variation in avian escape strategy across a sample of 96 species of birds. Brain mass, body size, habitat structure and group size were the main predictors of escape strategy. The direction of the effect of these traits was consistent with selection for a reduction of monitoring costs. Therefore, attentional costs depend on relative brain size, which determines the ability to monitor the whereabouts of potential predators and the difficulty of this task as reflected by habitat and social complexity. Thus brain size, and the cognitive functions associated with it, constitute a general framework for explaining the effects of body size, habitat structure and sociality identified as determinants of avian escape strategy.

After detecting an approaching predator, animals must decide when to flee. A seminal paper^1^ developed the economic logic behind such decisions and noted that animals should not simply flee immediately upon detecting a predator, but rather when the risks of remaining and the costs of flight are equal. Three decades of research supports this ‘economic escape theory^2^,^3^,^4^’. Hundreds of studies document the many factors affecting the optimal escape decision of prey^2^,^3^,^4^, such as body size, predation pressure, distance from refuge, habitat structure, immediate energy requirements, and engagement in social activities.

Perhaps counter-intuitively, however, recent studies have shown that, in most taxa, the distance in which prey becomes aware of and begins to monitor the predator (i.e. alert distance; AD) explains most variation in the decision of prey to flee^5^,^6^. Empirically, this is inferred by a strong positive relationship between AD and flight initiation distance (FID; the predator-prey distance when escape begins). In some cases^7^, such a relationship is so strong that none or very little variation remains to be explained by the myriad of factors that the economic escape literature has identified^2^,^3^,^4^.

The “Flush Early and Avoid the Rush” (FEAR) hypothesis aims to solve this paradox by stating that animals will flee an approaching predator soon after detection in order to minimise the costs incurred by monitoring an approaching predator^8^. Therefore, an early escape (inferred by a positive AD-FID relationship) is a consequence of this cost. The FEAR hypothesis does not contradict economic escape theory, but rather recognises a possible ubiquitous and disproportionally important cost neglected by it: the attentional costs associated with on-going monitoring of a potential predator^8^,^9^,^10^,^11^. The attentional cost is an opportunity cost because, once the prey must redirect its limited attention to monitoring predator, the prey’s ability to enjoy the benefits of delaying flight (e.g. engage in foraging, social activities or maintenance) is reduced, leading prey to respond optimally by fleeing early^9^.

Two key steps towards understanding the drivers of optimal escape decisions were taken recently. Once correlational statistics proved to be problematic to quantify how immediately prey flee after detection, the first important step was the development of a metric to directly infer the prey’s escape strategy, termed the phi (Φ) index^12^. Φ is a standardised goodness-of-fit metric that measures how close to AD FID is: Φ value ~1 means that individuals of a species flush significantly sooner after detection (which provides little opportunity to engage in fitness-enhancing activities), whereas as Φ-value decreases, individuals of a species tolerate a closer approach before flight (which provides a greater opportunity to engage in fitness-enhancing activities) (see Methods for additional details on Φ). The second step used both meta-analytic^5^ and comparative approaches^6^ to document species’ escape strategies. These analyses revealed that many, but not all, avian species flush significantly soon after detecting predators. Yet, there was variation in the degree in which species tend to flush early from predators^5^,^6^. The logical next step, therefore, is to understand the drivers of this variation in escape strategy among species. Which species-specific traits are correlated with prey’s escape strategies? How could these traits provide insights into the mechanisms underlying optimal escape decisions?

Here we provide a comprehensive evaluation of the drivers of species’ escape strategy by investigating the morphological, life history and natural history traits potentially related to escape strategy of 96 avian species (representing 74 genera from 48 families). Specifically, we predicted how variation in prey’s escape strategy (inferred by Φ) was correlated with brain mass, body mass, clutch size, use of habitat, group size, migratory behaviour, and capture of live prey.

Species that do not flush immediately upon detecting predators have the opportunity to benefit from fitness-enhancing activities, such as foraging or mating^1^,^13^. Brains also play a crucial role in behaviour^14^,^15^. If relative brain size reflects cognitive ability, then we should expect escape behaviour to correlate with relative brain size. Specifically, if large-brained species are better able to both maximize their benefits before flight and minimise their monitoring costs (e.g. through a better assessment of a predator’s distance and speed^16^,^17^), while escaping successfully, we expected that large-brained species would tend to flush later than small-brained species^11^. Body mass has been shown to explain considerable variation in risk-taking behaviour of many taxa^2^,^3^,^18^,^19^. We predicted that larger birds will flush earlier to reduce the higher predation risk caused by their conspicuousness, or because a flight at closer distance becomes particularly energetically costly as body size increases^20^. After controlling for body size, species that lay larger clutches may have to forage more, or more intensively, so as to acquire resources that they can invest in their offspring. We therefore predicted that mean clutch size of a species should influence their propensity to accept a greater risk by flushing later from predators after detection.

We also expected that the degree of habitat openness influences birds’ escape strategies. Species that forage in open habitats may be able to detect predators from a greater distance^3^, and thus be more likely to invest particularly in on-going monitoring. Alternatively, species in dense habitats may not have the luxury of increased monitoring after detection because the predator may suddenly vanish. If dense habitat obstructs the ability to engage in on-going monitoring, we might expect that species in dense habitats flush earlier than species in open habitats. Group size may affect escape strategies of birds in two orthogonal ways^21^,^22^,^23^, each providing insights regarding causal effects. If birds in larger groups tend to flush earlier than birds in smaller groups, one can infer a strategy that prioritizes risk reduction. Such a finding would be inconsistent with the attentional cost proposed by the FEAR hypothesis. By contrast, if birds in larger groups tend to flush later, it suggests that the dilution effect^21^ or the many eyes effect^23^ permits birds to maximize their net benefits before fleeing. This relationship would be consistent with the mechanism proposed by the FEAR hypothesis if increased collective vigilance by a large group reduces the *per capita* cost of monitoring. Migratory species live in different habitats with different predator communities at different times of the year. Hence, individuals of migratory species should monitor their surroundings more cautiously than residents that are familiar with the predator community on a daily basis. Finally, a previous study showed that species that eat live prey are more responsive to predators^18^, an effect possibly explained by a carry-over effect of having a better motion sensitive vision^24^,^25^. If so, we might expect the propensity to flee early after detection to be influenced by a species’ diet.

## Results

**Figure 1:**
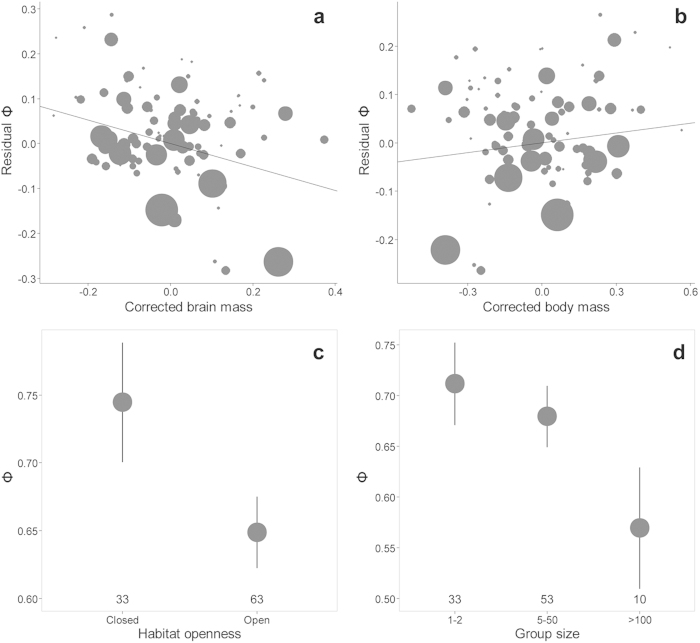
Effects of (a) brain mass, (b) body mass, (c) habitat openness, and (d) group size on interspecific escape strategy of birds. Escape strategy quantified by the phi index (Φ), an effect size metric that measures how immediately prey escape from predators upon detection. Larger Φ-values imply that prey escape at a distance close to the detection distance. Corrected (a) brain mass and corrected (b) body mass are residual values of these variables after controlling for their shared effect and different sizes of points reflect differences in a species’ sample size. Plots c and d show mean ± 95% confidence intervals; the number of species tested at each level is shown.

The minimum model retained four variables (*R*^2^ = 0.44), all with intermediate to large effect sizes: brain mass, body mass, habitat openness, and group size (full and minimum adequate models are presented in Table 1). The most important predictor of escape strategy was brain mass, with larger-brained species (after controlling for body mass) delaying escape from predators (Table 1, Fig. 1). In contrast, larger species flushed earlier from predators than smaller species (Table 1, Fig. 1). On average, species inhabiting closed habitats flushed earlier than species in open habitats (Table 1, Fig. 1). Finally, species allowed closer approach of predators as flock size increased (Table 1, Fig. 1). Our findings were robust to the use of alternative data and analyses (Supplementary Tables S1–S5).

## Discussion

The main findings of this study of escape strategy were that brain mass, body mass, habitat structure and flock size explained a large fraction of the variance among avian species. Escape strategy is hypothesised to depend on monitoring costs paid by prey for knowing the whereabouts of an approaching predator. Minimisation of such attentional costs depends on the relative size of the brain of prey that in turn determines the ability to monitor the predator and the difficulty of this task as reflected by habitat openness (i.e. visual environment) and flock size. These conclusions were independent of a number of potentially confounding variables.

Brain size and the cognitive functions associated with it constitute a general framework for explaining the effects of body size, habitat structure and sociality identified as determinants of escape strategy in this study. Brain mass was the single-most important predictor of Φ —an index of the degree to which a species tolerates approach before initiating flight. Brain size is hypothetically linked to the risk of predation through its effects on anti-predator defense, although such an effect has rarely been documented. Schulz and Dunbar^26^ showed a strong bias against large-brained prey in the diet of chimpanzees *Pan troglodytes* and felids, supposedly because such prey are particularly efficient at evading predators. A second study showed the relative brain size of ungulates was positively associated with whether a species was social or not and social complexity as reflected by group size and the number of individuals of the two sexes in breeding aggregations, as well as the use of more closed habitats^27^. This effect is consistent with brain size being linked to risk of predation and the probability of early detection of an approaching predator. Relative brain size and breeding sociality (i.e. number of breeding pairs in a particular site) were also linked in several orders of mammals, although such a link may relate to other selective forces than predation in these taxa^28^. There is even intraspecific evidence showing that relative brain size in barn swallows *Hirundo rustica* increases with colony size and the challenge of a complex social environment (e.g. a higher predation pressure caused by a higher aggregation of prey)^29^. Ungulates, carnivores and primates all showed that an increase in sociality was strongly correlated with an increase in relative brain size^30^. While the later study rested on the assumption that group cohesion is cognitively demanding, and that social conflicts may affect the ability of individuals to acquire basal resources^30^, predation risk may constitute yet another selective force of sufficient generality to affect the evolution of brain size.

An additional selective force that may covary with the evolution of brain size is the consumption of brains by predators. Both avian^31^ and mammalian^32^ predators are known to eat the brain of their prey. Indeed, brain tissue is one of the first parts consumed by these predators. Such a preference is most likely linked to the nutritional value of the brain. Any preferred food will be over-represented in the diet, and such a preference might result in selection for larger brains, which in turn might increase predation risk and select for cognitive abilities to outwit their predators. Future studies are required to test this hypothesized coevolutionary scenario.

Predation risk is strongly linked to the degree of sociality due to dilution effects^21^, selfish herd effects^22^ and the benefits of many eyes^23^. These mechanisms have since long been assumed to play a key role in the evolution of anti-predator behaviour^33^. Our findings that species tend to delay escape after detection of predators as group size increases, is consistent with reduction of attentional costs proposed by the FEAR hypothesis. Based on this finding, we can pool such behaviour into a single framework in which escape strategy has evolved in response to cognitive abilities and the complexity of undertaking such monitoring of predators in a given social and physical environment.

Large bodied species fled at greater distances and relatively sooner after detecting predators. We infer that this reduced the higher predation risk caused by their conspicuousness, or because a flight at closer distance becomes particularly energetically costly as body size increases^20^,^34^. This finding alone does not bear on the FEAR hypothesis but other results illustrate the important role that monitoring and detection play on shaping escape strategies.

The main predicted mechanism hypothesised to explain the flush early phenomenon is a monitoring cost imposed on prey in order to ascertain the whereabouts and the activities of the predator^8^. Such monitoring costs have been documented in several species implying that they impose costs by reducing ingestion rates or acquisition of other limiting resources^8^,^9^,^10^. In fact, using an independent data set of European birds, Møller and Erritzøe^11^ showed that flight initiation distance increased with relative eye size and decreased with relative brain size. We initially aimed to include eye size in these analyses but we were unable to find eye size measurements for most species of our data set; the effect of eye size thus remains an open question. However, a separate analysis using the mean distance at which a species detected the approaching threat (alert distance), as a surrogate for visual abilities, did not identify a significant effect of alert distance (see Supplementary Table S6). Interestingly, flight initiation distance increased with relative size of the cerebellum, which plays a key role in motor control. These findings suggest that cognitive monitoring of a potential predator is a key determinant of risk-taking behaviour.

These results suggest several future research directions. First, it would be interesting to monitor brain activity and heart rate of potential prey using remote sensing equipment while simultaneously recording the anti-predator behaviour discussed in the present study. This would allow us to verify the degree to which different portions of the brain are activated during risk assessment and escape. Such studies might be conceivably conducted in controlled captive situations that create a virtual reality from the subject’s perspective, although an effect of captivity should be considered (e.g. increase in docility of individuals). Second, it would be interesting to investigate what happens in terms of brain size evolution in predator-free oceanic islands. Thus, systematic studies of island-mainland comparisons would be very revealing. Third, degradation of habitats such as caused by industrial agriculture, forestry and fisheries is known to reduce quantity and quality of food, which in turn has negative effects on development of costly organs such as the brain^35^. Our study suggests that such effects may reduce the ability of individuals to monitor predators with negative consequences for predation risk. Finally, future studies can assess how the capacity for multi-modal risk assessment of prey^36^, such as visual ‘and’ acoustic cues, influence risk-taking.

In conclusion, we have shown that escape strategy, specifically a type of wariness, by different species of birds is related to the cost of monitoring a possible predator. Such costs are associated with the relative size of the brain that determines the ability to perform monitoring and the difficulty of this task as reflected by habitat and social complexity.

## Methods

### Field data collection

FID data were collected in United States and Australia from 1999 to 2005 using a standard protocol^18^,^37^. Data were collected throughout the year to avoid systematic effects potentially caused by season. Observers identified birds that were foraging or engaged in ‘relaxed behaviours’, such as roosting or preening. Highly vigilant, obviously alarmed, or nesting individuals were not approached, nor were endangered species. FID was measured by walking directly towards the subject at 0.5 m/s. Observers were previously trained to maintain speed constant while minimising excessive vertical movement across a variety of terrains^38^,^39^. A marker was dropped at the starting point of the approach. Subsequent flags were dropped when the animal first oriented itself towards the approaching human (alert distance; AD) and when the animal began to flee (flight initiation distance; FID). The distances between these markers were afterwards measured to the nearest 0.1 m. Observers attempted to avoid resampling individuals by flushing birds in different geographical locations and not resampling the same location repeatedly. A modest degree of resampling subjects, however, has been shown to not influence the results of studies like this^39^.

### Calculating the phi index

The relationship between AD and FID is constrained by an envelope; FID can only assume values equal to or lower than its actual AD (a prey cannot run away from a predator before it has detected it). Because of this envelope constraint, inferring escape strategy of species using correlational statistics on AD and FID may be inappropriate much of the time because such statistics might violate assumptions of the statistical test (heteroscedasticity), are particularly sensitive to outliers, and because they do not directly measure how immediately a species flee after predator detection, and thus their escape strategy^12^. Therefore, to correctly test how immediately a prey flees from a predator, we used the phi index (Φ), which is a non-parametric goodness-of-fit metric that measures how close to AD FID is^12^. We calculated Φ using the following equation:


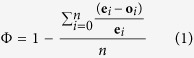


where, e_*i*_ is the AD, o_*i*_ is the FID, and *n* is the sample size. Importantly, Φ can be used as an effect size measure which provides the magnitude and direction of the effect of AD on FID^12^,^40^. Φ is a standardized metric, i.e. it ranges from 0 to 1. Φ-values that deviate from 0.5 (the null expectation; analogous to a Pearson’s *r* = 0 in non-constrained relationships) are a robust indication of a species that flushes later (<0.5, not consistent with FEAR prediction) or earlier (>0.5, consistent with the FEAR prediction).

### Covariates

Most information about brain mass comes from Iwaniuk and Nelson^41^, whereas missing information was completed with data from other sources^25^,^41^,^42^,^43^,^44^,^45^,^46^. High repeatability among studies indicates that information on brain mass can be combined across sources^47^. Data for the remaining six variables were collected from a single source^48^. The complete data set with information on covariates is available in Supplementary Data. Below we provide details about the covariates.

### Body mass

Measured as mean body mass (in grams). If body masses of males and females were provided separately, we used the average value for the species. In a separate model selection, we used the mean body mass data of individuals for which brain mass was actually measured to check if our results were sensitive to the body mass data used (Supplementary Table S5).

### Brain mass

Measured as mean brain mass (in grams).

### Clutch size

We used the mean clutch size of a species per reproductive period.

### Habitat openness

We coded species into two categories: species that forage in open habitat (e.g. uplands and grassland) or species that forages in closed habitats (e.g. dense forests and woodlands).

### Group size

We coded species into three categories: single or in pairs, in groups of 5–50 individuals, or groups containing >100 individuals.

### Migratory behaviour

We coded species as resident or migratory. In cases where migratory status of a species changed in function of their geographical location, we relied on information of the populations actually studied to assert their migratory behavior (south-eastern populations from Australia, and, respectively, southern and western populations from California and Colorado, United States).

### Capture of live prey

We coded species as species that capture live prey or species that do not capture live prey.

### Statistical methods

Because there was an absence of phylogenetic structure in the residuals of our statistical models (see Supplementary Methods), we fitted Ordinary Least Squares (OLS) models using Φ as the response variable and body mass, brain mass, clutch size, habitat openness, group size, migratory behaviour, and capture of live prey as the independent predictor variables. All models were weighted by sample size to account for differences in sampling effort among species^49^,^50^,^51^. Continuous variables were log_10_ transformed before analyses to achieve normality. Visual inspection of residuals showed that our models matched the assumptions of homoscedasticity and normality required by OLS regressions^52^.

As expected, brain mass was strongly positively correlated with body mass (*r *= 0.95). However, multiple regression is the best approach to control for undesirable confounding effects among correlated covariates, yielding unbiased coefficient estimates^53^. For this reason, we retained body mass and brain mass in the same model to control for their confounded effect. The remaining predictor variables presented low multicollinearity (variance inflation factor, VIF < 1.62, below the threshold of 3^52^).

We performed stepwise backward model selection based on corrected Akaike Information Criteria (AICc), using a threshold value of 2 (conclusions were the same using a stepwise selection based on *P*-values with a threshold of 0.05 or 0.1). We present both full and minimal adequate models. We assessed the importance of each predictor based on effect sizes calculated as partial correlation coefficients, at which quantifies the effect of a given predictor while controlling for the effects of other covariates included in the model^54^. We followed criteria listed by Cohen^55^ for small (*r *= 0.10, explaining 1% of the variance), intermediate (*r *= 0.3, explaining 9% of the variance) or large effect sizes (*r *= 0.5, explaining 25% of the variance). All analyses were conducted with R^56^.

### Ethics statement

Field data collection was approved by Macquarie University Animal Care Committee (protocol # 99021) and the University of California Los Angeles Animal Research Committee (IACUC # 2000-147-01), and the methods were carried out in accordance with the approved guidelines. Data were collected on public and private land after acquiring any required permits. By design, experimental approaches were designed to create only a brief disturbance and we are not aware of any lasting harm caused by the experimental approaches. In addition, and to reduce the likelihood of any negative effects, endangered species were not targeted, and we only targeted birds away from their nests. In fact, the disturbance produced to birds by our methodology did not differ from standard “background” disturbance caused by any persons walking outdoors.

## Additional Information

**How to cite this article**: Samia, D. S. M. *et al.* Brain size as a driver of avian escape strategy. *Sci. Rep.* **5**, 11913; doi: 10.1038/srep11913 (2015).

## Supplementary Material

Supplementary Information

Supplementary Data 1

## Figures and Tables

**Table 1:** Full and minimum adequate models to explain interspecific variation in escape strategy (Φ) of birds.

Predictor	Level	Estimate	SE	*t*	*P*	Effect size
Full model (AICc = −148.2, *R*^2^ = 0.45 )						
(Intercept)		0.494	0.097	5.11	**<0.001**	
Body mass		0.147	0.055	2.62	**0.01**	0.26
Brain mass		−0.355	0.090	−3.94	**<0.001**	0.38
Habitat openness		0.076	0.027	2.78	**0.007**	0.28
Group size	5–50 individuals	−0.052	0.022	−2.32	**0.023**	0.23
	>100 individuals	− 0.097	0.034	−2.85	**0.005**	0.28
Clutch Size		0.039	0.056	0.71	0.479	0.07
Capture of live prey		−0.028	0.026	−1.07	0.286	0.11
Migratory behaviour		0.019	0.024	0.79	0.429	0.08
Minimal model (AICc = −153.1, *R*^2^ = 0.44)						
(Intercept)		0.469	0.091	5.19	**<0.001**	
Body mass		0.162	0.052	3.14	**0.002**	0.31
Brain mass		−0.378	0.083	−4.54	**<0.001**	0.42
Habitat openness		0.083	0.026	3.19	**0.002**	0.31
Group size	5–50 individuals	−0.053	0.021	−2.39	**0.019**	0.24
	>100 individuals	−0.081	0.031	−2.53	**0.013**	0.25

Effect sizes are partial correlation coefficients. *P*-values in bold indicate significance (*P *< 0.05).
