# Supplementary material for: Brain size as a driver of avian escape strategy
Source: Sci Rep. 2015 Jul 3;5:11913. doi: 10.1038/srep11913 (PMC4490409; doi:10.1038/srep11913)
Supplement: Supplementary Information [file srep11913-s1.pdf]

Supplementary Information

Brain size as a driver of avian escape strategy

Diogo S. M. Samia[1*], Anders Pape Møller[2], Daniel T. Blumstein[3]

[1]Laboratory of Theoretical Ecology and Synthesis, Department of Ecology, Federal University of Goiás, CP. 131, 74001-970 Goiânia, Brazil, [2]Laboratoire d'Ecologie, Systématique et Evolution, CNRS UMR 8079, Université Paris-Sud, Bâtiment 362, F-91405 Orsay Cedex, France, [3]Department of Ecology and Evolutionary Biology, University of California, 621 Young Drive South, Los Angeles, California, 90095-1606, USA

Correspondence and requests for materials should be addressed to D.S.M.S. (diogosamia@gmail.com).

Supplementary Methods

Testing for phylogenetic signal in residuals

Closely related species are more likely to have similar phenotypes because of their shared ancestry, which may result in data points being statistically dependent by producing more similar residuals than expected by chance from an OLS regression[1]. A commonly used technique to account for such phylogenetic structure in residuals is the Phylogenetic Generalised Least Squares (PGLS) regression[2]. However, the use of PGLS in the absence of a phylogenetic signal in residuals is to be avoided because it can

produce biased coefficient estimates and substantially increase the Type I error rate[3,4]. We tested if there was phylogenetic signal in the residuals of our comparative model by using Pagel's λ[5,6] as implemented in the R package "phytools"[7]. A λ-value that does not differ significantly from 0 indicates absence of phylogenetic signal in the residuals (justifying the use of non-phylogenetic statistical models, such as OLS), while a λ-value that differs significantly from 0 indicates some phylogenetic signal in residuals (demanding application of phylogenetically-informed models, such as PGLS)[8]. We used a recent phylogenetic avian hypothesis for our analyses[9] (Supplementary Fig. S1).

Phylogenetic Generalised Least Squares models

Although our models did not have significant phylogenetic structure in the residuals (see Supplementary Results), we opted to fit PGLS models to test for robustness of our results. We fitted PGLS models assuming two different evolutionary scenarios: (1) assuming that the trait evolved under a Brownian motion model of evolution (optimizing the Pagel's λ parameter[10]), and (2) assuming that the trait evolved under an Ornstein-Uhlenbeck (OU) model of evolution (optimizing the α parameter[11]). PGLS models were fitted using the "gls" function of the R package "nlme"[12]. The models were weighted by sample size to account for differences in sampling effort among species[4,13,14]. To do so, we used the inverse of the sample size in the variation function structure (argument "weights" of the "gls" function[15]). We used two phylogenetic trees[9] in our models to test if our conclusions were sensitive to the choice of phylogeny: the Ericson backbone and the Hackett backbone phylogenies (Supplementary Fig. S1).

Supplementary Results

There was no phylogenetic signal in the residuals of either of our candidate models ($\lambda < 0.36$, $P > 0.41$), indicating that Ordinary Least Squares (OLS) regressions was the appropriate model to fit (results shown in the main text). However, PGLS models yielded exactly the same conclusions as OLS models. Full and minimum adequate PGLS models assuming different models of evolution and using both phylogenies are shown in Supplementary Tables S1-S4. Finally, our conclusions were qualitatively similar when we replaced the current body mass data from our OLS models by the body mass data of individuals for which brain mass was actually measured (r between body mass data = 0.997; Supplementary Table S5).

Figure S1. Phylogenetic hypotheses based on Ericson and Hackett backbone for the 96 avian species included in the present study.

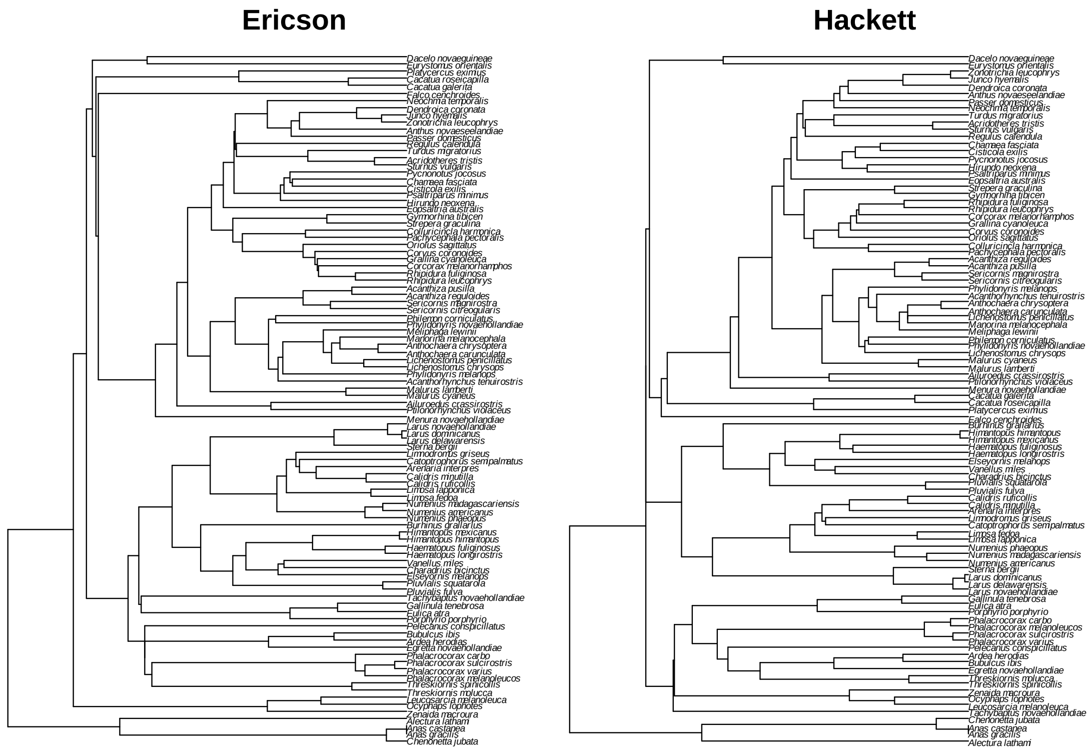

Supplementary Table S1. Full and minimum adequate PGLS model to explain interspecific variation in escape strategy (Φ) of birds based on the Ericson phylogeny and assuming a Brownian motion model of evolution. Effect sizes are partial correlation coefficients. *P*-values in bold indicate significance ($P < 0.05$). The optimised phylogenetic parameter (Pagel's λ) of each model is shown.

Predictor	Level	Estimate	SE	*t*	*P*	Effect size
Full model (AICc = -148.8, λ = 0.35)						
(Intercept)		0.417	0.121	3.44	**<0.001**	
Body mass		0.174	0.068	2.57	**0.0119**	0.26
Brain mass		-0.383	0.103	-3.73	**<0.001**	0.36
Habitat openness		0.070	0.027	2.62	**0.010**	0.26
Group size	5 – 50 individuals	-0.052	0.022	-2.34	**0.022**	0.23
	> 100 individuals	-0.093	0.032	-2.85	**0.005**	0.28
Clutch Size		0.025	0.058	0.42	0.673	0.04
Capture of live prey		-0.034	0.029	-1.16	0.248	0.12
Migratory behaviour		0.022	0.026	0.85	0.396	0.09
Minimal model (AICc = -153.8, λ = 0.36)						
(Intercept)		0.389	0.115	3.39	**0.001**	
Body mass		0.182	0.064	2.85	**0.005**	0.28
Brain mass		-0.394	0.097	-4.05	**<0.001**	0.39
Habitat openness		0.077	0.025	3.07	**0.003**	0.30
Group size	5 – 50 individuals	-0.051	0.021	-2.40	**0.019**	0.24
	> 100 individuals	-0.078	0.031	-2.54	**0.013**	0.25

Supplementary Table S2. Full and minimum adequate PGLS model to explain interspecific variation in escape strategy (Φ) of birds based on the Hackett phylogeny and assuming a Brownian motion model of evolution. Effect sizes are partial correlation coefficients. *P*-values in bold indicate significance (*P* < 0.05). The optimised phylogenetic parameter (Pagel's λ) of each model is shown.

Predictor	Level	Estimate	SE	*t*	*P*	Effect size
Full model (AICc = -148.5, λ = 0.31)						
(Intercept)		0.409	0.121	3.37	**0.0011**	
Body mass		0.179	0.067	2.65	**0.0094**	0.26
Brain mass		-0.393	0.102	-3.84	**<0.001**	0.37
Habitat openness		0.070	0.027	2.65	**0.010**	0.26
Group size	5 – 50 individuals	-0.050	0.022	-2.25	**0.027**	0.23
	> 100 individuals	-0.091	0.033	-2.78	**0.007**	0.28
Clutch Size		0.027	0.058	0.46	0.646	0.05
Capture of live prey		-0.031	0.028	-1.08	0.285	0.11
Migratory behaviour		0.022	0.026	0.83	0.409	0.09
Minimal model (AICc = -153.7, λ = 0.32)						
(Intercept)		0.382	0.114	3.34	**0.0012**	
Body mass		0.189	0.063	2.98	**0.004**	0.29
Brain mass		-0.406	0.096	-4.21	**<0.001**	0.40
Habitat openness		0.077	0.025	3.08	**0.003**	0.30
Group size	5 – 50 individuals	-0.049	0.021	-2.31	**0.023**	0.23
	> 100 individuals	-0.077	0.031	-2.48	**0.015**	0.25

Supplementary Table S3. Full and minimum adequate PGLS model to explain interspecific variation in escape strategy (Φ) of birds based on the Ericson phylogeny and assuming an Ornstein-Uhlenbeck model of evolution. Effect sizes are partial correlation coefficients. *P*-values in bold indicate significance ($P < 0.05$). The optimised phylogenetic parameter (α) of each model is shown.

Predictor	Level	Estimate	SE	*t*	*P*	Effect size
Full model (AICc = -97.4, α = 59.7)						
(Intercept)		0.498	0.097	5.15	**<0.001**	
Body mass		0.145	0.056	2.60	**0.011**	0.26
Brain mass		-0.354	0.090	-3.95	**<0.001**	0.38
Habitat openness		0.076	0.027	2.79	**0.007**	0.28
Group size	5 – 50 individuals	-0.058	0.022	-2.67	**0.009**	0.27
	> 100 individuals	-0.098	0.034	-2.90	**0.005**	0.29
Clutch Size		0.036	0.056	0.65	0.520	0.07
Capture of live prey		-0.025	0.027	-0.91	0.366	0.09
Migratory behaviour		0.021	0.024	0.86	0.394	0.09
Minimal model (AICc = -117.9, α = 77.8)						
(Intercept)		0.474	0.090	5.27	**<0.001**	
Body mass		0.160	0.051	3.12	**0.002**	0.31
Brain mass		-0.377	0.083	-4.56	**<0.001**	0.43
Habitat openness		0.083	0.026	3.20	**0.002**	0.31
Group size	5 – 50 individuals	-0.056	0.021	-2.67	**0.009**	0.27
	> 100 individuals	-0.081	0.032	-2.57	**0.012**	0.26

Supplementary Table S4. Full and minimum adequate PGLS model to explain interspecific variation in escape strategy (Φ) of birds based on the Hackett phylogeny and assuming an Ornstein-Uhlenbeck model of evolution. Effect sizes are partial correlation coefficients. *P*-values in bold indicate significance ($P < 0.05$). The optimised phylogenetic parameter (α) of each model is shown.

Predictor	Level	Estimate	SE	*t*	*P*	Effect size
Full model (AICc = -97.4, α = 8.7)						
(Intercept)		0.498	0.097	5.15	**<0.001**	
Body mass		0.145	0.056	2.60	**0.011**	0.26
Brain mass		-0.354	0.090	-3.95	**<0.001**	0.38
Habitat openness		0.076	0.027	2.79	**0.007**	0.28
Group size	5 – 50 individuals	-0.058	0.022	-2.67	**0.009**	0.27
	> 100 individuals	-0.098	0.034	-2.90	**0.005**	0.29
Clutch Size		0.036	0.056	0.65	0.520	0.07
Capture of live prey		-0.025	0.027	-0.91	0.366	0.09
Migratory behaviour		0.021	0.024	0.86	0.394	0.09
Minimal model (AICc = -118.0, α = 11.3)						
(Intercept)		0.474	0.090	5.27	**<0.001**	
Body mass		0.160	0.051	3.12	**0.002**	0.31
Brain mass		-0.377	0.083	-4.56	**<0.001**	0.43
Habitat openness		0.083	0.026	3.20	**0.002**	0.31
Group size	5 – 50 individuals	-0.056	0.021	-2.67	**0.009**	0.27
	> 100 individuals	-0.081	0.032	-2.57	**0.012**	0.26

Supplementary Table S5. Full and minimum adequate Ordinary Least Squares model to explain interspecific variation in escape strategy (Φ) of birds using mean body mass from individuals used to measure brain mass. Effect sizes are partial correlation coefficients. *P*-values in bold indicate significance ($P < 0.05$).

Predictor	Level	Estimate	SE	*t*	*P*	Effect size
Full model (AICc = -147.2, R^2 = 0.45)						
(Intercept)		0.514	0.096	5.36	**<0.001**	
Body mass		0.138	0.057	2.42	**0.0178**	0.24
Brain mass		-0.348	0.095	-3.68	**<0.001**	0.36
Habitat openness		0.073	0.027	2.66	**0.009**	0.26
Group size	5 – 50 individuals	-0.055	0.023	-2.38	**0.020**	0.24
	> 100 individuals	-0.095	0.035	-2.75	**0.007**	0.27
Clutch Size		0.040	0.057	0.71	0.482	0.07
Capture of live prey		-0.028	0.027	-1.03	0.305	0.11
Migratory behaviour		0.023	0.024	0.97	0.337	0.10
Minimal model (AICc = -153.9, R^2 = 0.43)						
(Intercept)		0.486	0.091	5.36	**<0.001**	
Body mass		0.156	0.053	2.93	**0.004**	0.29
Brain mass		-0.377	0.088	-4.26	**<0.001**	0.40
Habitat openness		0.080	0.026	3.07	**0.003**	0.30
Group size	5 – 50 individuals	-0.053	0.022	-2.41	**0.018**	0.24
	> 100 individuals	-0.076	0.032	-2.38	**0.020**	0.24

Supplementary Table S6. Full and minimum adequate Ordinary Least Squares model to explain interspecific variation in escape strategy (Φ) of birds using mean alert distance as an additional predictor. Effect sizes are partial correlation coefficients. *P*-values in bold indicate significance ($P < 0.05$). There was no phylogenetic signal in the residuals of either of candidate models ($\lambda < 0.36$, $P > 0.41$).

Predictor	Level	Estimate	SE	*t*	*P*	Effect size
Full model (AICc = -146.2, R^2 =0.46)						
(Intercept)		0.476	0.101	4.72	**<0.001**	0.44
Body mass		0.125	0.065	1.93	0.0567	0.20
Brain mass		-0.345	0.092	-3.76	**<0.001**	0.36
Habitat openness		0.084	0.030	2.81	**0.006**	0.28
Group size	5 – 50 individuals	-0.053	0.023	-2.31	**0.023**	0.23
	> 100 individuals	-0.097	0.034	-2.83	**0.006**	0.28
Clutch Size		0.037	0.056	0.66	0.510	0.07
Capture of live prey		-0.032	0.027	-1.17	0.247	0.12
Migratory behavior		0.019	0.024	0.79	0.435	0.08
Alert distance		0.046	0.071	0.65	0.519	0.07
Minimal model (AICc = -153.1, R^2 = 0.44)						
(Intercept)		0.46835	0.091	5.19	**<0.001**	
Body mass		0.16194	0.052	3.14	**0.002**	0.31
Brain mass		-0.37831	0.083	-4.54	**<0.001**	0.42
Habitat openness		0.08303	0.026	3.19	**0.002**	0.31
Group size	5 – 50 individuals	-0.05247	0.021	-2.39	**0.019**	0.24
	> 100 individuals	-0.08067	0.031	-2.53	**0.013**	0.25

Supplementary References

1. Felsenstein, J. *Inferring Phylogenies*. (Sinauer Associates, Inc., 2004).

2. Grafen, A. The phylogenetic regression. *Philos. Trans. R. Soc. London B* **326,** 119–157 (1989).

3. Revell, L. J. Phylogenetic signal and linear regression on species data. *Methods Ecol. Evol.* **1,** 319–329 (2010).

4. Garamszegi, L. Z. *Modern phylogenetic comparative methods and their application in evolutionary biology: concepts and practice*. (Springer, 2014).

5. Pagel, M. Inferring evolutionary processes from phylogenies. *Zool. Scr.* **26,** 331–348 (1997).

6. Pagel, M. Inferring the historical patterns of biological evolution. *Nature* **401,** 877–84 (1999).

7. Revell, L. J. phytools: an R package for phylogenetic comparative biology (and other things). *Methods Ecol. Evol.* **3,** 217–223 (2012).

8. Symonds, M. R. E. & Blomberg, S. P. A primer on phylogenetic generalised least squares. In: *Modern phylogenetic comparative methods and their application in evolutionary biology: concepts and practice* (ed Garamszegi, L. Z.) 105–130 (Springer, 2014).

9. Jetz, W., Thomas, G. H., Joy, J. B., Hartmann, K. & Mooers, A. O. The global diversity of birds in space and time. *Nature* **491,** 444–448 (2012).

10. Freckleton, R. P., Harvey, P. H. & Pagel, M. Phylogenetic analysis and comparative data: a test and review of evidence. *Am. Nat.* **160,** 712–726 (2002).

11. Butler, M. A. & King, A. A. Phylogenetic comparative analysis: a modeling approach for adaptive evolution. *Am. Nat.* **164,** 683–695 (2004).

12. Pinheiro, J., Bates, D., DebRoy, S. & Sarkar, D. Linear and nonlinear mixed effects models. *R Packag. v. 3.1-111* (2011).

13. Garamszegi, L. Z. & Møller, A. P. Effects of sample size and intraspecific variation in phylogenetic comparative studies: a meta-analytic review. *Biol. Rev.* **85,** 797–805 (2010).

14. Garamszegi, L. Z. & Møller, A. P. Nonrandom variation in within-species sample size and missing data in phylogenetic comparative studies. *Syst. Biol.* **60,** 876–80 (2011).

15. Paradis, E., Claude, J. & Strimmer, K. S. APE: Analyses of Phylogenetics and Evolution in R language. *Bioinformatics* **20,** 289–290 (2004).
